# Integrative Analysis of lncRNA and mRNA and Profiles in Postoperative Delirium Patients

**DOI:** 10.3389/fnagi.2021.665935

**Published:** 2021-05-19

**Authors:** Yuxiang Song, Xiaoyan Wang, Aisheng Hou, Hao Li, Jingsheng Lou, Yanhong Liu, Jiangbei Cao, Weidong Mi

**Affiliations:** ^1^Medical School of Chinese PLA, Beijing, China; ^2^Department of Anesthesiology, The First Medical Center of Chinese PLA General Hospital, Beijing, China; ^3^Department of Anesthesiology, The Fourth Medical Center of Chinese PLA General Hospital, Beijing, China

**Keywords:** postoperative delirium, long non-coding RNAs, microarray, co-expression, competing endogenous RNA

## Abstract

Delirium is a common serious complication that often occurs after major surgery. The goals of this study were to explore the expression profiles and functional networks of long non-coding RNAs (lncRNAs) and mRNAs in patients of postoperative delirium (POD). Microarray analysis was performed on the peripheral blood samples to identify differentially expressed (DE) lncRNAs and mRNAs in 4 POD patients and 4 non-POD volunteers. DE lncRNAs and mRNAs were validated by quantitative reverse transcription PCR (RT-qPCR). Bioinformatic analyses were performed to identify the critical biological functions and signaling pathways involved in POD. A total of 1195 DE lncRNAs and 735 DE mRNAs were identified between the POD and non-POD groups. Verified by the RT-qPCR, we identified 14 DE lncRNAs that may relate to the pathogenesis of POD. These 14 DE lncRNAs play important regulatory roles in “glutamate and 5-hydroxytryptamine,” “synaptotagmin 7,” “transient receptor potential channel,” “interleukin-2 production.” There was a regulatory relationship between lncRNA ENST00000530057 and synaptotagmin (Syt) 7 mRNA. The mRNA level of PCLO was up-regulated in POD group. This study showed abundant DE lncRNAs and mRNAs in POD that might help in deciphering the disease pathogenesis.

## Introduction

Delirium is a common serious complication that often occurs after major surgery. The incidence of delirium in people over 60 years of age after major surgery is approximately 20-50% (Dasgupta and Dumbrell, 2006; Avidan et al., 2017). Since many elderly people need to undergo surgery, postoperative delirium (POD) has become a growing public health problem and a major challenge to our rapidly growing aging population. POD usually takes place within the first 24-48 postoperative hours. It is characterized by an acutely altered mental state, manifested as acute brain dysfunction, fluctuating level of consciousness, disorganized thinking and inattention. This represented acute brain dysfunction (Subramaniyan and Terrando, 2019). Delirium is usually one of the most painful events experienced by patients and their families during the perioperative period. In addition, POD is associated with increased mortality, cognitive and functional decline, prolonged hospital length of stay, and significant increases in medical expenses. POD even leads to long-term cognitive impairment or even permanent dementia (Skrobik, 2011). Because of the grave nature and heavy burden of delirium, people have come up to improve their understanding of delirium. Scientists are working to prevent POD and reduce its impact prevent POD and alleviate its impact (Vlisides and Avidan, 2019). However, the pathophysiology of delirium is still not fully understood, which seriously affects the management and prevention of POD.

Non-coding RNA was regarded as transcript noise in past decades. Recently, studies have revealed that non-coding RNA was involved in various diseases (Zhang et al., 2019; Fulzele et al., 2020; Guo Z. et al., 2020; Kaarniranta et al., 2020). Long non-coding RNAs (lncRNAs) are a class of RNA molecules with more than 200 nucleotides in length (Li et al., 2013). Although they are not involved in encoding proteins, lncRNAs directly regulate various transcriptional, epigenetic and post-transcriptional protein encodings in the form of RNA (Guo et al., 2019). LncRNAs are involved in various normal brain functions and diseases, including aging (Catana et al., 2020), psychiatric and neurodegenerative illnesses such as Parkinson’s disease (PD) (Zhao et al., 2019; Huaying et al., 2020), major depressive disorder, autism, and schizophrenia (Briggs et al., 2015; Parikshak et al., 2016; Liu Y. et al., 2018; Zhou et al., 2018). lncRNA-T199678 mitigated the α-Syn-induced dopaminergic neuron injury via targeting miR-101-3p, which contributed to promote PD (Bu et al., 2020). lncRNAs have been found to participate in the pathogenesis related to PD by regulating neuroinflammation, oxidative stress (Bu et al., 2020), cell cycle, mitochondrial dysfunction, and amyloid β (Aβ) production (Chen Y. et al., 2019; Gao et al., 2020). In addition, lncRNA Xist regulates osteoblast differentiation by sponging miR-19a-3p in aging-induced osteoporosis of bone marrow mesenchymal stem cells (Chen et al., 2020). A recent profiling study also showed that many lncRNAs were significantly altered in peripheral blood of depressed patients (Cui et al., 2017). Meng et al. (2018) found that lncRNA DGCR5 regulates many schizophrenia-related genes in human neural progenitor cells derived from human induced pluripotent stem cells. The expression of lncRNA NON-HSAT089447 was increased in peripheral blood of schizophrenic patients. NON-HSAT089447 participated in dopamine signaling pathway through upregulating DRDs (Chen S. et al., 2019). However, the roles of lncRNAs in POD have not been extensively explored.

In this study, the expression changes of lncRNAs and mRNAs in the peripheral blood from elderly patients who had developed delirium after orthopedic surgery were evaluated through microarray analysis. The functional enrichment analysis and annotation were carried out to explore the roles of lncRNAs in POD. The findings of this study provide a new insight into the roles of lncRNAs in POD and pave the road for further investigations of the underlying functions and regulatory mechanisms of lncRNAs in POD.

## Materials and Methods

### Patients and Blood Sample Collection

Peripheral blood samples from 4 old adults with POD after orthopedic surgery and 4 non-POD after orthopedic surgery (>75 years) were obtained at Chinese PLA General Hospital. Control subjects were non-POD volunteers after orthopedic surgery with age and gender matched to those of the POD cases. The peripheral blood samples were collected between June 2020 and August 2020. All POD cases were clinically diagnosed according to the POD diagnosis criteria. Control subjects were non-POD volunteers after orthopedic surgery with age and gender matched to those of the POD cases. The baseline demographic summary of the POD group and non-POD group is shown in Tables 1, 2. This study was approved by the Ethics Committee of Chinese PLA General Hospital (Beijing, China) (No. S2017-096-02). All patients provided written informed consent.

**TABLE 1:** Characteristics of the Patients.

No.	Age	Gender	BMI	Surgery	Surgery duration (min)	Anesthesia	CHD	CVD	Hypertension	DM	Delirium
1	84	woman	27.5	Lumbar spine surgery	140	GA	no	no	no	no	yes
2	81	woman	26.7	Lumbar spine surgery	100	GA	no	no	no	no	no
3	83	man	25.7	Humerus fractures	155	GA	no	yes	no	no	yes
4	81	man	23.4	Lumbar spine surgery	170	GA	no	no	yes	no	no
5	93	woman	23.7	Femoral neck fracture	190	NB	yes	yes	yes	yes	yes
6	87	woman	19.5	Fracture of right femoral neck	70	NB	no	no	no	no	no
7	93	woman	20.8	Right femoral intertrochanteric fracture	80	GA	no	yes	no	no	yes
8	86	woman	18.7	Proximal fracture of left humerus	120	GA	no	yes	yes	yes	no

*Anesthesia: GA = general anesthesia, NB = nerve block anesthesia; CHD: coronary heart disease; DM: diabetes mellitus; CVD: Cerebrovascular Disease.*

**TABLE 2:** Demographic characteristics between POD and non-POD patients.

Characteristics	POD (*n* = 4)	Non-POD (*n* = 4)	P*
Age	88.25 (4.76)	83.75 (2.77)	0.207
Gender, male, n (%)	1 (25)	1 (25)	1.000
BMI	24.45 (2.50)	22.09 (3.19)	0.353
Surgery duration (min)	141.3 (39.75)	115 (36.40)	0.431
CHD, n (%)	1 (25)	0 (0)	0.285
CVD, n (%)	3 (75)	1 (25)	0.157
Hypertension, n (%)	1 (25)	2 (50)	0.465
DM, n (%)	1 (25)	1 (25)	1.000

*P values are for comparison between POD and non-POD group. The P value was calculated with the use of the Mann-Whitney U for compare continuous variables. The P value was calculated with the use of Fisher Chi-squared test for categorical variables. Abbreviations: BMI: Body Mass Index, CHD: Coronary Heart Disease, CVD: Cardiovascular Disease, DM: Diabetes Mellitus.

### Microarray Hybridization and Analysis

Microarray hybridization was carried out by Aksomics (Shanghai, China). Blood total RNA was extracted and purified using TRIzol Reagent (Invitrogen, United States) and RNasey Mini Kit (Qiagen, German) from POD group (*n* = 4) and non-POD group (*n* = 4). The quantity and purity of total RNA samples were measured by NanoDrop ND-1000 (ThermoFisher, United States). RNA was further amplified and labeled by Quick Amp Labeling Kit, One-Color (Agilent, United States), purified and hybridized with an ArrayStar Human lncRNA microarray V5.0 (ArrayStar, United States). Data were extracted with Feature Extraction version version 11.0.1.1 (Agilent, United States). Raw data were normalized by the Quantile algorithm, limma packages in R. After quantile normalization of the raw data, lncRNAs and mRNAs for which at least 4 out of 8 samples had flags of Present or Marginal (“All Targets Value”) were chosen for further data analysis. The statistical significance of differentially expressed lncRNAs and mRNAs among groups (POD vs. non-POD) was identified by the cutoff of 1.5-fold change and *P* < 0.05 and displayed by volcano plot filtration. Hierarchical clustering was carried out to show the distinguishable DE lncRNAs and DE mRNAs expression patterns among samples (Faghihi and Wahlestedt, 2009; Shi and Shang, 2016).

### Quantitative Real-Time PCR

To validate our microarray data, quantitative real-time PCR experiments were performed using uperScriptTM III Reverse Transcriptase (Invitrogen, United States) and 2 × SYBR Green PCR Master Mix (Arraystar, United States) according to the manufacturer’s instructions. All data were normalized to β-actin data to calculate the relative concentrations of lncRNAs and mRNAs. Details of the genes and primers were listed in Supplementary Table 1.

### Functional Enrichment Analysis

Gene Ontology (GO) and Kyoto Encyclopedia of Genes and Genomes (KEGG) analysis were performed based on GO and KEGG database (Ashburner et al., 2000; Kanehisa et al., 2016) to obtain significant enriched GO terms and important biological functions involved in DE mRNAs and targeted mRNAs predicted by coding and non-coding co-expression (CNC) and competing endogenous RNA (ceRNA) networks. GO analysis was performed to explore the functional roles of DE mRNAs in terms of biological process (BP), cellular component (CC) and molecular function (MF). GO analysis was performed using database^1^. KEGG analysis was performed using the KEGG database^2^. The statistical significance of the GO and KEGG analysis enrichment were calculated by Fisher Exact test *P*-value and also −log10(p) transformed as the enrichment score. The recommend *P*-value was cut off less than 0.05.

### Protein-Protein Interaction (PPI) Network Analysis

Protein and protein interaction (PPI) analysis was performed based on STRING database^3^. Upregulated and downregulated mRNAs were used to construct the PPI network by Cytoscape (V3.6.0), respectively. And hub genes were obtained by screening the degree of connectivity of each node in the network. To identify the significant modules in the network, Cytoscape plugin Molecular Complex Detection (MCODE) were conducted with a score >4 in up-regulated DE mRNAs and a score > 5 in down-regulated DE mRNAs.

### DE lncRNAs-DE mRNAs Interaction Analysis

To reveal the potential regulatory relationships between the targeted DE lncRNAs and DE mRNAs, targeted DE lncRNAs co-expressed with DE mRNAs were identified. The significant DE lncRNA–DE mRNA co-expression pairs were identified by Pearson correlation coefficients values | r | ≥ 0.9 and *P*-values of no less than 0.005. In addition, to identify DE lncRNAs nearby DE mRNAs with cis−regulatory effects, DE lncRNAs transcribed within a 200 kb window up or downstream of DE lncRNAs in POD and non-POD groups were identified.

### Targeted DE lncRNAs Associated ceRNA Network Construction

The potential miRNA response elements were identified within the sequences of targeted DE lncRNAs and DE mRNAs. We used miRanda^4^ and TargetScan database^5^ to search the potential miRNA-binding sites. Then, lncRNA-miRNA-mRNA network was constructed based on lncRNA-miRNA and miRNA-mRNA regulation pairs. In addition to overlapping miRNA binding, mRNAs with an expression pattern in the same direction as lncRNAs were filtered out to construct lncRNA-miRNA-mRNA ceRNA networks by Cytoscape.

### Statistical Analysis

Normality of data was tested using the Shapiro-Wilk normality test. Log of probe signal values of each group were consistent with normal distribution. Data were expressed as mean ± SD and were analyzed by Student’s t test analysis of variance by GraphPad Prism software version 5.0. *P* < 0.05 was considered significant differences.

## Results

### LncRNAs & mRNAs Microarray

**FIGURE 1:**
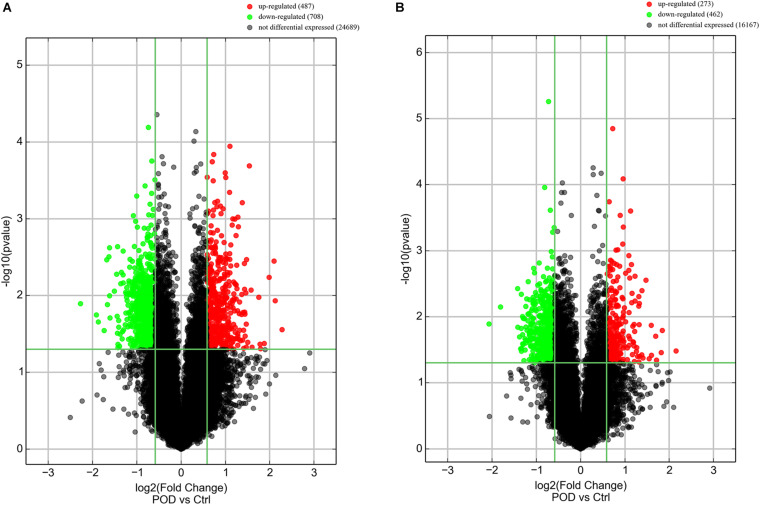
The changes in expression profiling of lncRNAs **(A)** and mRNAs **(B)** in POD group compared with non-POD group. POD, postoperative delirium.

**FIGURE 2:**
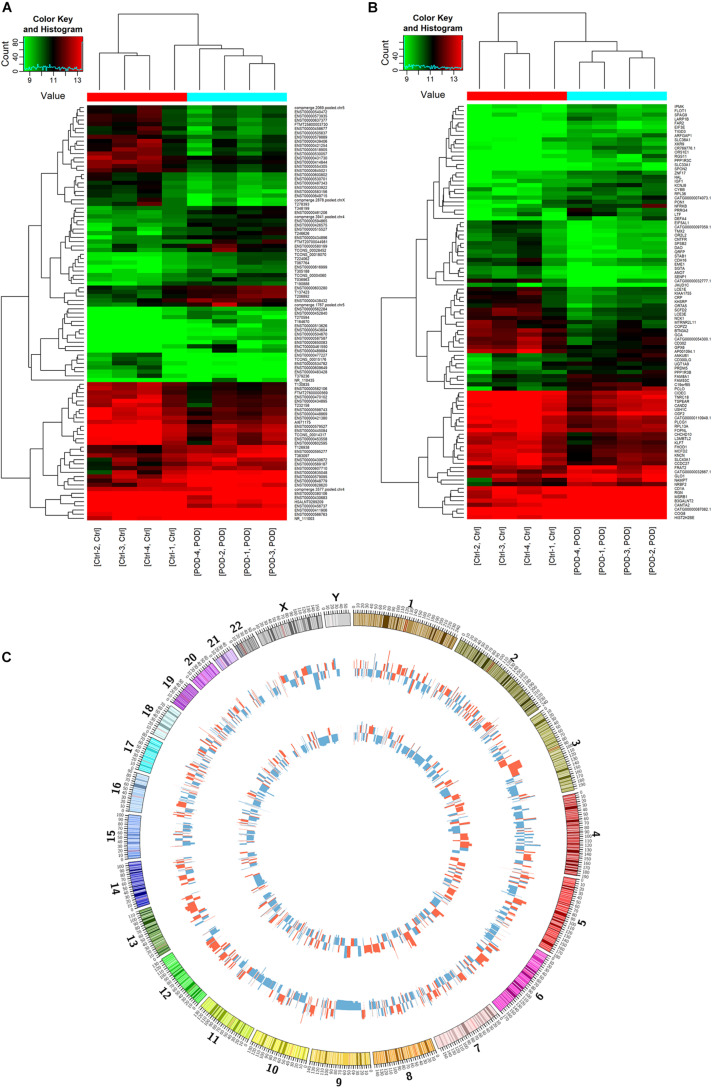
Identification of DE lncRNAs and mRNAs. Heat map of top 50 expressed DE lncRNAs **(A,B)** DE mRNAs between POD and non-POD group. **(C)** DE lncRNAs and DE mRNAs circos plots. The 1-th inner circle is a histogram plot of chromosome. The 2-nd inner circle is a histogram plot of the significant differences lncRNA, circle with red color are up-regulated lncRNA, circle with blue color are down-regulated lncRNA. The 3-rd inner circle is a histogram plot of the significant differences mRNA, circle with red color are up-regulated mRNA, circle with blue color are down-regulated mRNA. POD, postoperative delirium; DE, differentially expressed.

The results of this study showed that the expression of 487 lncRNAs and 273 mRNAs were significantly up-regulated, the expression of 708 lncRNAs and 462 mRNAs were significantly down-regulated in the POD group compared with the non-POD group (Figures 1A,B). The top 20 DE lncRNAs and DE mRNAs are shown in Supplementary Tables 2, 3. The Top 50 DE lncRNAs and DE mRNA were chosen for cluster analysis based to their fold change in expression level, respectively (Figures 2A,B). Circos plots representing the distribution of DE lncRNAs and DE mRNAs on chromosomes are displayed in Figure 2C. The data supporting the findings of this study are openly available in the NCBI GEO database under the accession numbers GSE163943.

### Functional Annotation of DE mRNAs

**FIGURE 3:**
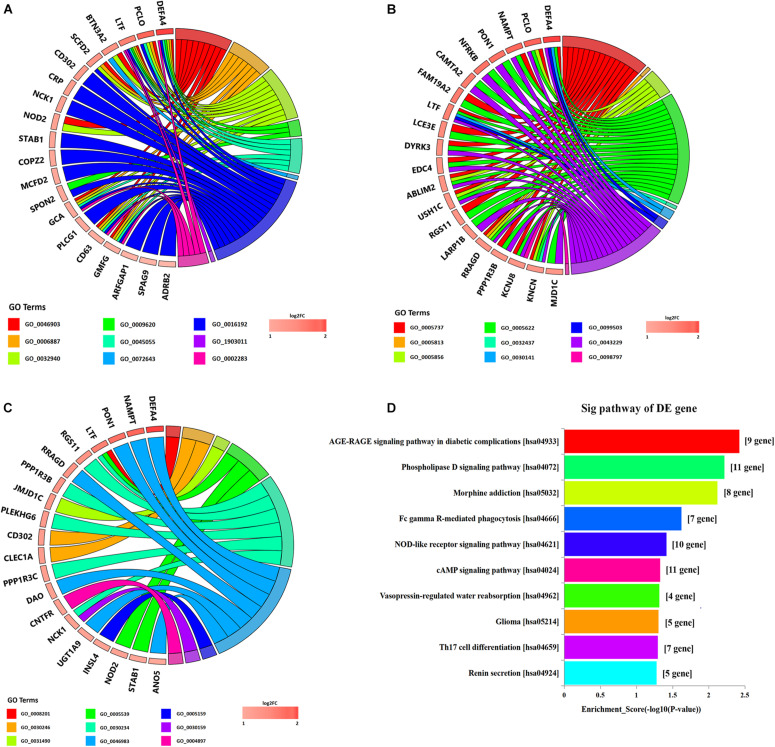
Gene Ontology and Kyoto Encyclopedia of Genes and Genomes pathway analyses of differentially expressed mRNAs in POD and non-POD group. **(A)** biological processes GO terms. **(B)** cellular components GO terms. **(C)** molecular function GO terms. **(D)** KEGG pathways. Fisher exact test, *P* < 0.05. POD, postoperative delirium; GO, Gene Ontology; KEGG, Kyoto Encyclopedia of Genes and Genomes.

Gene Ontology and KEGG pathways were employed to analyze DE mRNAs. GO analysis showed that the most significantly enriched GO terms were positive regulation of gamma-delta T cell activation (BP), intracellular organelle (CC), and lipoprotein lipase activator activity (MF). The top 10 GO terms of DE mRNAs are shown in Figures 3A-C and Supplementary Tables 4, 5. KEGG analysis of the DE mRNAs showed that the significantly enriched pathways were AGE-RAGE signaling pathway in diabetic complications, phospholipase D signaling pathway, morphine addiction, Fc gamma R-mediated phagocytosis, NOD-like receptor signaling pathway, etc. The top 10 KEGG pathways of DE mRNAs are shown in Figure 3D and Supplementary Tables 6, 7.

### Protein and Protein Interaction Analysis of DE mRNAs

**FIGURE 4:**
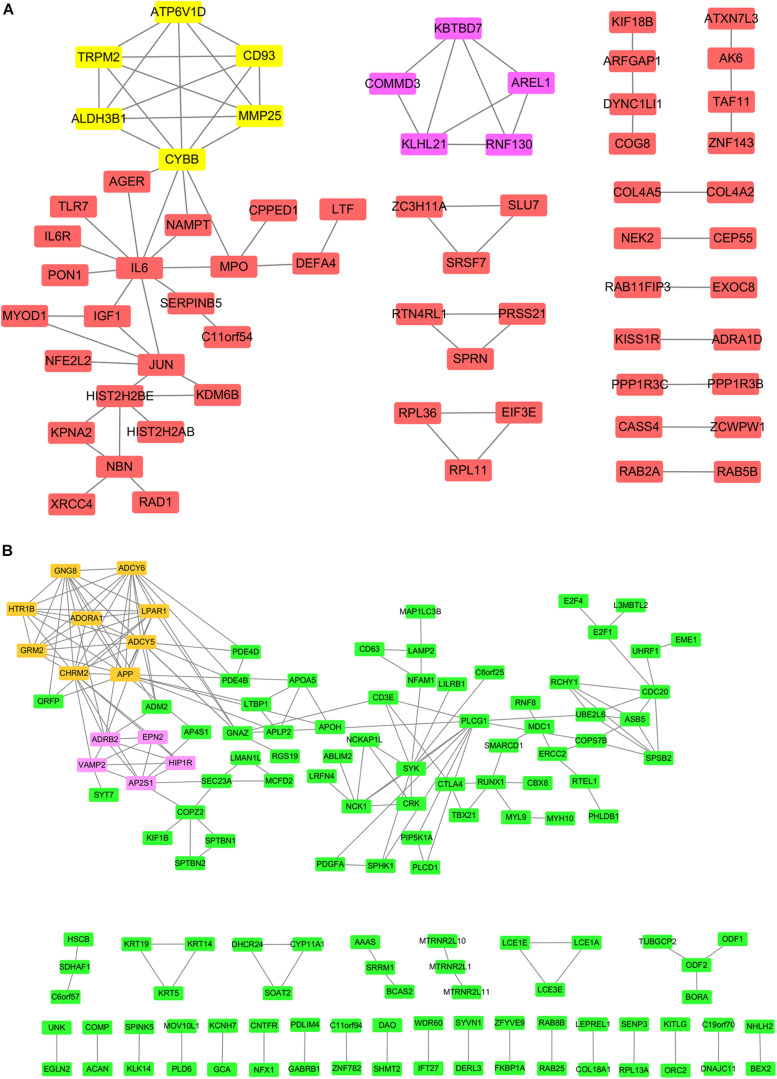
The protein–protein interaction (PPI) network of top 200 up-regulated and down-regulated DE mRNAs. **(A)** PPI network top 200 up-regulated DE mRNAs. Red circles indicate up-regulated genes. Yellow and purple circles indicate 2 modules. **(B)** PPI network top 200 down-regulated DE mRNAs. Green circles indicate down-regulated genes. Orange and purple circles indicate 2 modules. DE, differentially expressed.

Protein and protein interaction analysis of the significantly up- and down-regulated mRNAs was conducted separately. PPI network was constructed by cytoscape (V3.6.0). These results suggested that the network of up-regulated mRNAs contained 65 nodes and 75 edges (average of 2.308 candidates, minimum required interaction score ≥0.7), and the network of down-regulated mRNAs were composed of 132 nodes and 189 edges (average of 2.864 neighbors, minimum required interaction score ≥0.7) (Figures 4A,B). Screening hub genes that had no less than 5 neighboring genes produced two significant modules with MCODE score >4 in significantly up-regulated mRNAs (Figure 4A) and two significant modules with MCODE score >5 in significantly down-regulated mRNAs (Figure 4B).

### Validation of lncRNA and mRNA Microarray Results Using RT-qPCR

**FIGURE 5:**
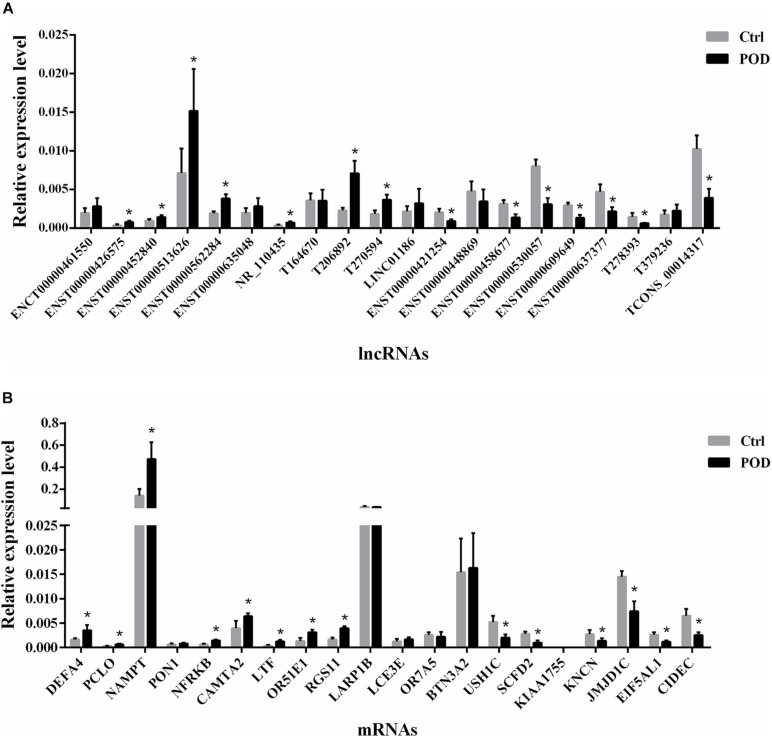
Validation of lncRNAs and mRNAs expression by RT-qPCR. **(A)** The relative expression levels of selected lncRNAs detected by the RT-qPCR. **(B)** The relative expression levels of selected mRNAs detected by the RT-qPCR. β-Actin was used as a housekeeping gene for normalizing changes in specific gene expression. *P < 0.05 vs non-POD group, n = 4.

To validate our microarray results, we selected 20 DE lncRNAs (10 up-regulated and 10 down-regulated lncRNAs) and 20 DE mRNAs (10 up-regulated and 10 down-regulated mRNAs) for RT-qPCR experiments. Up-regulated and down-regulated DE lncRNAs and DE mRNAs that were filtered out by the criteria of fold change >2, *P* < 0.05 and raw intensity >500. Last, we selected the top 10 up-regulated and top 10 down-regulated DE lncRNAs and DE mRNAs according to fold change for RT-qPCR experiments. The results showed that among the 20 DE lncRNAs, 7 lncRNAs were significantly up-regulated, 7 lncRNAs were significantly down-regulated, and the expression pattern of 6 lncRNAs did not change significantly (Figure 5A). Among the 20 DE mRNAs, 8 mRNAs were significantly up-regulated, 6 mRNAs were significantly down-regulated, and the expression pattern of 6 mRNAs was not significantly changed (Figure 5B). Compared with the microarray results, the validation rate of both DE lncRNAs and DE mRNAs were 70%.

### Target DE lncRNAs-DE mRNAs Co-expression Network

**FIGURE 6:**
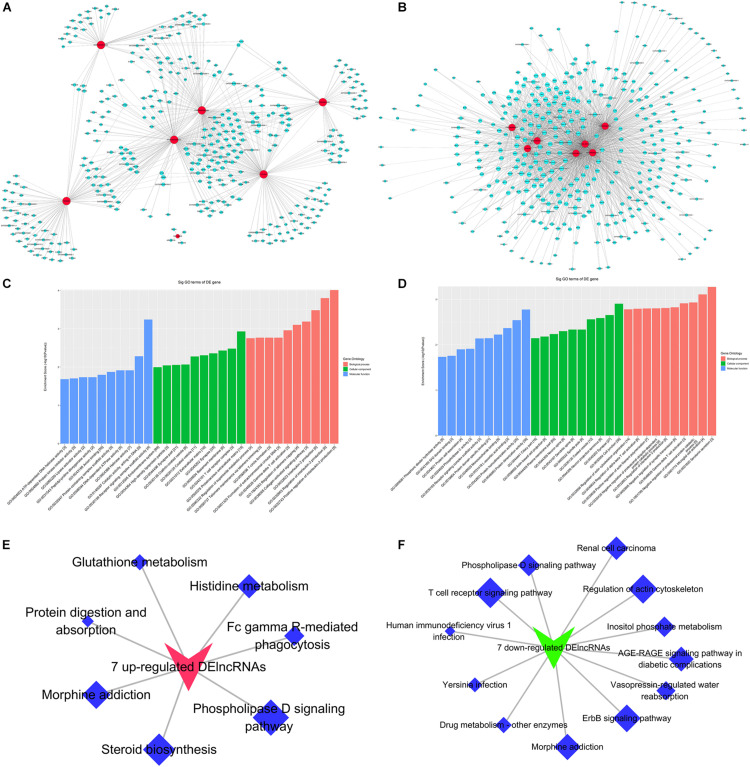
Target DE lncRNAs-DE mRNAs co-expression network. **(A)** CNC network of 7 up-regulated lncRNAs-DE mRNAs **(B)** CNC network of 7 down-regulated lncRNAs-DE mRNAs Red nodes are lncRNAs; Blue nodes are mRNAs. Positive correlation is a solid line, negative correlation is a dashed line. | r | ≥ 0.9, P < 0.005. **(C)** Top 10 GO terms of GO analysis based on up-regulated CNC analysis results. **(D)** Top 10 GO terms of GO analysis based on down-regulated CNC analysis results. **(E)** Top 10 pathways of KEGG pathway analysis based on up-regulated CNC analysis results. **(F)** 7 Top 10 pathways of KEGG pathway analysis based on down-regulated CNC analysis results. Red node indicate 7 up-regulated lncRNAs; Green node indicate 7 down-regulated lncRNAs; Blue node indicates KEGG Pathways. CNC, coding and non-coding co-expression; DE, differentially expressed.

Based on the results of quantitative validation, we performed co-expression analysis and constructed CNC network. We incorporated 7 up-regulated and 7 down-regulated lncRNAs and 735 DE mRNAs (273 significantly up-regulated and 462 significantly down-regulated mRNAs), respectively, with the Pearson correlation coefficient | r | ≥ 0.9 and *P* < 0.005 as the screening criteria. As a result, we obtained 639 matched lncRNA-mRNA pairs for 7 up-regulated lncRNAs and 735 DE mRNAs. Among them 190 lncRNA-mRNA pairs were positively modulated and 449 lncRNA-mRNA pairs were negatively modulated. The network consisted of 373 nodes and 639 edges (Figure 6A). For 7 down-regulated lncRNAs and 735 DE mRNA, 1586 matched lncRNA-mRNA pairs were identified, including 1426 lncRNA-mRNA pairs that were positively modulated and 160 lncRNA-mRNA pairs that were negatively modulated. The network contained 383 nodes and 1586 edges (Figure 6B).

Next, we conducted GO and KEGG pathway analyses of DE mRNAs co-expressed with 7 up-regulated lncRNAs and 7 down-regulated lncRNAs. The GO analysis showed that DE mRNAs co-expressed with 7 up-regulated lncRNAs were mainly enriched in positive regulation of interleukin-2 production in BP terms, proteinaceous extracellular matrix in CC terms, and receptor signaling complex scaffold activity in MF terms (Figure 6C). DE mRNAs co-expressed with 7 down-regulated lncRNAs were mainly enriched in serotonin secretion in BP terms, cell junction in CC terms, and protein dimerization activity in MF terms (Figure 6D). The results of KEGG analysis showed that the 7 up-regulated lncRNAs might be involved mainly in the pathways, including phospholipase D signaling pathway, steroid biosynthesis, morphine addiction, etc. (Figure 6E). The 7 down-regulated lncRNAs might be involved in T-cell receptor signaling pathway, regulation of actin cytoskeleton, rbB signaling pathway, etc (Figure 6F).

### DE lncRNA-Nearby DE mRNA Interaction Network

**FIGURE 7:**
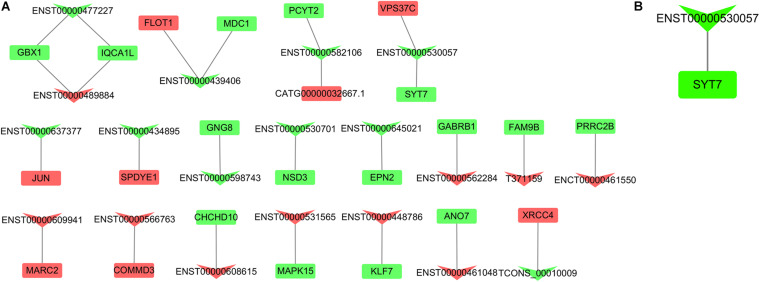
Top 20 DE lncRNA-nearby DE mRNA interaction network. **(A)** DE lncRNA-nearby DE mRNA interaction network. **(B)** Interaction network showing the overlap of the DE lncRNA-DE mRNA co-expression network with the DE lncRNA-nearby DE mRNA interaction network. Rectangle and inverted triangles represent DE mRNAs and DE lncRNAs, respectively. Red and green colors indicate up- and down-regulation, respectively. DE, differentially expressed.

To understand the biological function of DE lncRNAs, we performed nearby gene prediction and obtained 163 DE lncRNA-nearby target DE mRNA pairs that consisted of 130 DE lncRNAs (45 up-regulated lncRNAs, 85 down-regulated lncRNAs) and 150 DE mRNAs (55 up-regulated mRNAs, 95 down-regulated mRNAs). The top 20 up-regulated and down-regulated lncRNAs and their nearby target DE mRNAs from 130 DE lncRNAs were selected to construct the network (Figure 7A and Table 3). As a result, a pair of lncRNA-mRNA, namely lncRNA ENST00000530057 and mRNA synaptotagmin (Syt) 7 (Figure 7B) was obtained by looking for the overlap in DE lncRNA-DE mRNA co-expression network and DE lncRNA-nearby DE mRNA interaction network.

**TABLE 3:** Detail information of Top 20 up-regulated and down-regulated DE lncRNAs and their nearby genes.

lncRNA	Gene Symbol	*P-* -Value lncRNA	Regulation lncRNA	Genome Relationship	Nearby Gene	*Nearby Gene Symbol*	*P-* -*value mRNA*	Regulation mRNA
ENST00000562284	AC107398.3	0.023566706	up	upstream	ENST00000295454	GABRB1	0.006502478	Down
ENCT00000461550	CATG00000109 981.1	0.047027038	up	upstream	ENST00000357304	PRRC2B	0.039760245	down
T371159	G087517	0.048720122	up	upstream	ENST00000327220	FAM9B	0.018125736	down
ENST00000609941	AL445423.1	0.044475755	up	downstream	ENST00000366913	MARC2	0.002246648	up
ENST00000489884	FASTK	0.036630864	up	upstream	ENST00000297537	GBX1	0.041211942	down
ENST00000489884	FASTK	0.036630864	up	upstream	ENST00000615129	IQCA1L	0.001887133	down
ENST00000566763	AL158211.1	0.01445867	up	downstream	ENST00000376836	COMMD3	0.004404543	up
ENST00000608615	AP000345.2	0.001208178	up	upstream	ENST00000401675	CHCHD10	0.022970992	down
ENST00000531565	BREA2	0.041337699	up	downstream	ENST00000338033	MAPK15	0.01163593	down
ENST00000448786	AC007879.3	0.014133393	up	downstream	ENST00000309446	KLF7	0.014330277	down
ENST00000461048	SEPT2	0.006597391	up	upstream	ENST00000274979	ANO7	0.017201782	down
ENST00000530057	TKFC	0.028598773	down	downstream	ENST00000540677	SYT7	0.009616245	down
ENST00000530057	TKFC	0.028598773	down	upstream	ENST00000301765	VPS37C	0.021366888	up
TCONS_00010009	XLOC_004448	0.003126583	down	upstream	ENST00000511817	XRCC4	0.010632905	up
ENST00000637377	AL136985.3	0.005275579	down	downstream	ENST00000371222	JUN	0.027857069	up
ENST00000434895	MYL7	0.018636373	down	downstream	ENST00000258704	SPDYE1	0.029287705	up
ENST00000477227	NOS3	0.021154249	down	downstream	ENST00000297537	GBX1	0.041211942	down
ENST00000477227	NOS3	0.021154249	down	downstream	ENST00000615129	IQCA1L	0.001887133	down
ENST00000439406	HCG20	0.03674178	down	upstream	ENST00000376389	FLOT1	0.006667504	up
ENST00000439406	HCG20	0.03674178	down	upstream	ENST00000376406	MDC1	0.009149454	down
ENST00000582106	MAFG-DT	0.005379508	down	downstream	FTMT26800005164	CATG0000 0032667.1	0.013118419	up
ENST00000582106	MAFG-DT	0.005379508	down	upstream	ENST00000538721	PCYT2	0.005145985	down
ENST00000598743	AC093503.1	0.006842395	down	upstream	ENST00000300873	GNG8	0.023894996	down
ENST00000530701	FGFR1	0.006910915	down	downstream	ENST00000317025	NSD3	0.040803072	down
ENST00000645021	B9D1	0.004271313	down	downstream	ENST00000314728	EPN2	0.0132114	down

### Targeted DE lncRNAs Associated ceRNA Network Construction

**FIGURE 8:**
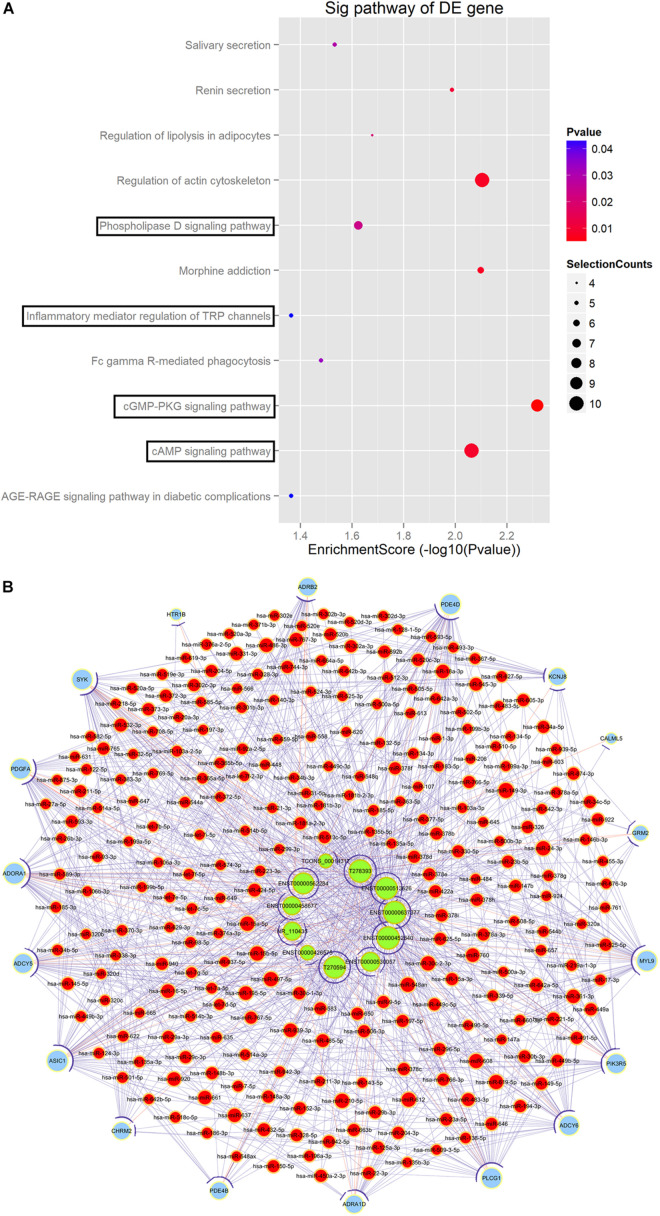
Targeted DE lncRNAs associated ceRNA network construction. **(A)** KEGG analysis based on ceRNA network. **(B)** Targeted DE lncRNAs associated ceRNA network. Fisher *t*-test. *P* < 0.05. Green nodes indicate DE lncRNAs. Red nodes indicate microRNAs. Blue nodes indicate DE mRNAs. ceRNA, competing endogenous RNA. DE, differentially expressed.

LncRNAs regulate mRNAs expression by competitively binding to miRNAs (competing endogenous RNA, ceRNA) is one of the main ways for lncRNAs to exert biological regulatory functions. We predicted the miRNA binding sites in 14 DE lncRNAs and 735 DE mRNAs by miRNA ID < 1000 based on TargetScan and miRanda database. We also performed KEGG pathway analysis of the predicted DE mRNAs (Figure 8A). The results suggested that four pathways, namely cGMP-PKG signaling pathway, cAMP signaling pathway, phospholipase D signaling pathway and inflammatory mediator regulation of TRP channels were associated with POD. We screened 11 DE lncRNAs, 18 DE mRNAs and 251 miRNAs to construct ceRNA network, using the genes enriched in the four pathways above, in combination with the predicted results of ceRNA from 14 DE lncRNAs and 735 DE mRNAs (Figure 8B).

## Discussion

In this study we analyzed the expression profiles of lncRNAs and mRNAs in the peripheral blood of four elderly patients who developed delirium after orthopedic surgery. The results suggested that the expression of 487 lncRNAs was up-regulated, and 708 lncRNAs were down-regulated. This suggested that lncRNA is very likely to be involved in the pathophysiology of POD. RT-qPCR validation showed that 14 DE lncRNAs were consistent with the microarray results. Therefore, we performed an in-depth functional analysis of these 14 DE lncRNAs in this study. The up-regulated ones are as the followings: ENST00000426575, ENST00000452840, ENST00000513626, ENST00000562284, NR_110435, T206892, T270594; and the down- regulated one are as the followings: ENST00000421254, ENST00000458477, ENST00000530057, ENST00000609649, ENST00000637377, T278393, T379236, CONS_00014317.

Glutamate is the main excitatory neurotransmitter in the central nervous system (CNS) and induces most of excitatory neurotransmissions in mammalian CNS (Mei et al., 2018). 5-hydroxytryptamine (5-HT), i.e., serotonin, is an additional important transmitter that regulates arousal, mood and cognition. Glutamate and 5-HT play an important role in cognitive function and in the development of neurodegenerative diseases. Too much and too little glutamate and 5-HT are both harmful because of the plasticity of the nervous system. Guo et al. found that serum glutamate levels decreased significantly in the group of elderly hip fracture patients with POD (Guo et al., 2017). Han et al. found that elderly hip fracture patients who developed POD had glutamate elevated in the preoperative cerebrospinal fluid (Han et al., 2020). Studies have found that 5-HT levels decrease in hyperexcitable delirium and increase in hypoexcitable delirium (Maldonado, 2013). Although a growing number of neurotransmitter changes have been identified in patients with POD, but the regulatory molecular mechanism remains largely unexplored. We performed KEGG analysis of the findings from the CNC network analysis and found that “glutamate metabolism” was associated with 7 regulatory lncRNAs which were the most significantly up-regulated. We also performed GO analysis of the findings from CNC network analysis and identified that “serotonin secretion” (BP term) was associated with 7 regulatory lncRNAs which were down-regulated most significantly. These indicated that lncRNAs plays an important role in the levels of some neurotransmitters in postoperative patients. These changes of lncRNA may be involved in the pathophysiology of POD. From these results, we speculated that these 14 validated DE lncRNAs might the major regulatory molecules and deserve further in-depth investigation into their functions.

The release of neurotransmitter is gained through the process of exocytosis (Südhof, 2013). A rise in intracellular Ca^2+^ triggers exocytosis (Südhof, 2012). Therefore, Ca^2+^ concentration is an important factor in the release of neurotransmitter (Xiao et al., 2017). Syt proteins can serve as calcium signaling sensors in the process of exocytosis (Xiao et al., 2017). There are 17 different isoforms in mammalian Syt family, of which Syt-7 has the highest Ca^2+^ sensitivity. Syt-7 is widely and highly expressed in the brain (Li et al., 2017). Wu et al. (2017) found that Syt-7 acts as a Ca^2+^ sensor in long term potentiation, mediating the exocytosis of Ca^2+^-dependent α-amino-3-hydroxy-5-methyl-4-isoxazole propionic acid receptors, thereby mediating memory and learning. The results of our microarray analysis suggested that in the peripheral blood of the POD group, ENST00000530057 was much less contained than that in the non-POD group (fold change = 3.3302004, *P* = 0.029), and the expression levels of Syt-7 mRNA were significantly lower than that in the non-POD group (fold change = 1.6525333, *P* = 0.009). DE lncRNA−nearby DE mRNA interaction network showed that there was a regulatory relationship between lncRNA ENST00000530057 and Syt-7 mRNA. This suggests that Syt-7 expression has been decreased in patients with POD, ENST00000530057 may contribute to regulate the expression level of Syt-7.

Furthermore, our KEGG analysis revealed that the ceRNA network was significantly enriched with the inflammatory mediator regulation of transient receptor potential (TRP) channels. TRP proteins work as ion channels with high Ca^2+^ permeability. TRP channels are involved in a variety of physiological processes (Li, 2017). Modulating TRP channels activity is an important pathway that affects cell function by regulating intracellular calcium levels (Dietrich et al., 2006). TRP family has seven members: TRPA (ankyrin), TRPC (canonical), TRPN (Drosophila NOMPC) (Li, 2017), TRPML (mucolipin), TRPM (melastatin), TRPP (polycystin), and TRPV (vanilloid). TRPC3, 4, 5, 6 are found in many regions of the brain (Zhou et al., 2008). TRPV1 is expressed in most neurons. TRPC4 has been shown to be involved in the neuronal exocytosis, regulation of neurite outgrowth and response to neural injury (Zechel et al., 2007). TRPC5 is closely related to fear-related behaviors and amygdala function (Riccio et al., 2009). Dysfunctions of TRP channels caused by alterations in levels of protein expression or regulatory factors were implicated in the occurrence of many diseases, including brain diseases. Currently, the research on TRP channels has aroused great interest in the pharmaceutical industry. As potential medications, agonists and antagonists of TRP channels are currently under development (Moran, 2018). TRPV1 antagonist was being developed for osteoarthritic pain. Unfortunately, it has been reportedly causes elevated body temperature in clinical trials (Krarup et al., 2011). Our research suggested that POD was associated with TRP channels altered by these 14 DE lncRNAs through the ceRNA network mechanism, where the likely-to-be-involved miRNAs were predicted. Our findings provide new ideas for looking at TRP channels as potential therapeutic targets.

In recent years, the impact of systemic inflammation on the brain has gained growing attention. Increasing evidence shows that blood-borne factors and the proinflammatory systemic environment have adverse effects on CNS function and directly affect cognitive function. Surgical trauma stimulated immune signal response, including monocyte activation. An imbalance between plasma levels of pro- and anti-inflammatory cytokines may be a predisposing factor for the development of POD in elderly patients (de Castro et al., 2014). Circulating neutrophils, monocytes, and other peripheral system factors can lead to alterations in glial homeostasis, synaptic plasticity and neuronal function (Reyes et al., 1999). Interleukin (IL)-6, tumor necrosis factor-α, and IL-1β are cytokines that trigger neuroinflammation in rodent postoperative models (Terrando et al., 2010). A large number of cytokine alterations are occurring in the patient’s peripheral blood (Cerejeira et al., 2012). Higher levels of CSF proinflammatory IL-6 are being used to predict cognitive decline after coronary artery bypass surgery (Kálmán et al., 2006; Capri et al., 2014). Studies have shown that the aberrant expression of lncRNA is also closely associated with the occurrence and development of systemic inflammation and neuroinflammation. LncRNAs frequently reported to regulate to cell apoptosis, angiogenesis and inflammation by has been be involved in post-stroke neuroprotection (Liang et al., 2020). LncRNAs also played a pivotal role in the regulation of vascular smooth muscle cells phenotype, functions, perhaps in the development of vascular aging related heart diseases (Ni et al., 2020). Regulation of cell apoptosis, angiogenesis and inflammation by lncRNAs has been frequently reported to be involved in post-stroke neuroprotection (Qian et al., 2020). These studies suggested that lncRNAs is involved in the process of inflammation. Our CNC network showed that the 7 up-regulated lncRNAs are significantly enriched in “positive regulation of IL-2 production”, “regulation of IL-2 production”, “IL-2 production” (BP terms). This result suggested that these 7 up-regulated lncRNAs may regulate IL-2 production. Sarinnapha et al. found that IL-6 levels measured on postoperative day 2 and IL-2 levels measured preoperatively and on postoperative day 2 were elevated in POD group compared to no-delirium controls (Vasunilashorn et al., 2015, 2019). However, Miriam et al. found that High IL-6 and low IL-2 levels were significantly associated with POD (Jiang and Lu, 1998; Capri et al., 2014). Miriam considered that IL-2 might have neuromodulatory effects, such as stimulating oligodendrocyte proliferation and maturation, promoting neuronal cell survival, analgesic effects, and possibly stimulating hypothalamic-pituitary axis. All these findings suggested that IL-2 is involved in the development of POD, although the results of these studies are different. The results of our study suggested that lncRNAs are important mechanisms that regulate IL-2 levels in the development of POD. The 7 up-regulated lncRNAs that we employed for prediction may be important regulatory molecules that merit further study.

Previous research found that there are a large number of mRNA changes in various brain diseases (Tao et al., 2019; Bucher et al., 2020; Guo X. et al., 2020; Jiang et al., 2020; Wang et al., 2020; Yang et al., 2020; Yuan et al., 2020; Zhu et al., 2020, 2021). Transcriptomic profiles from mRNA sequencing of peripheral blood with urinary tract infection patients showed that all patients with delirium had significant complement system activation compared to non-delirious patients (Kalantar et al., 2018). Xiang et al. processed for mRNA transcriptome analysis of hippocampus after tibial fracture surgery in C57BL/6J mice. Inflammatory mediator regulation of TRP channels, neuroactive ligand-receptor interaction and cholinergic synapse were overrepresented during the acute presence of hippocampal inflammation (Xiang et al., 2019). Illendula et al. (2020) found that many key proteins for the regulation of synaptic vesicle trafficking, neurotransmitter release and synaptic plasticity-were significantly down-regulated in old mice under surgery, anesthesia and intensive care environment induce delirium. Lipoprotein-associated phospholipase A2 and superoxide dismutase were independent risk factors of cognitive impairment in cerebral small vessel disease, and may be useful for the rapid evaluation of cognitive impairment in cerebral small vessel disease (Zhu et al., 2019). Weissleder et al. (2019) found that insulin like growth factor family members are local regulators of neurogenesis and indicate that the age-related reduction in insulin like growth factor 1 mRNA may limit new neuron production by restricting neuronal differentiation in the human subependymal zone. Yang et al. (2019) revealed an increase in Nwd1 expression in brain tissues from TLE patients, suggesting that this molecule may be associated with human epilepsy (Yang et al., 2019). In our study, our microarray analysis of mRNAs showed that 273 mRNAs were up-regulated and 462 mRNAs were down-regulated in the POD group. We found that the expression of presynaptic cytomatrix protein (PCLO) mRNA was significantly up-regulated, which was consistent with RT-qPCR validation (fold change = 3.5910908, *P* = 0.016). Previous research did not find this change of PCLO. PCLO plays an important role in monoaminergic neurotransmission in the brain. PCLO was identified as a gene overexpressed in the nucleus accumbens of mice repeatedly given methamphetamine, which may trigger severe mental disorders. The C-allele (risk allele) of single-nucleotide polymorphism rs2522833 within the PCLO gene plays a role in the pathophysiology of major depressive disorder (Igata et al., 2017). In this study, we found that significantly up-regulated PCLO expression might associate with the development of POD. The mechanism by which PCLO is up-regulated and the roles that PCLO plays in POD should be addressed in further research.

Postoperative delirium is the most common neurological complication in elderly patients after major surgery. There are numerous hypotheses to explain the pathogenesis of POD, such as the hypotheses of neurotransmitters imbalance, intraoperative hypoxia, acetylcholine deficiency, inflammatory impairment and so on. Unfortunately, the pathophysiological mechanisms of POD still remain unclear, and the regulatory factors leading to POD are poorly understood. Recent studies have found that lncRNAs play important roles in neuroinflammation and neurodegenerative disorders. Alzheimer’s disease (AD) is characterized by age-related neurodegenerative disorders and studies have found that it is regulated by non-coding RNA (Sarkar et al., 2019; Liu et al., 2020). 3,158 lncRNAs were identified of 629 patients with AD by microarray re-annotation in GEO database and the Affymetrix Human Genome U133 Plus 2.0 Array chip platform (Huaying et al., 2020). In Alzheimer’s disease rat model, up-regulation of lncRNA MEG3 can inhibit the activation of astrocytes in hippocampus in Alzheimer’s disease by inhibiting PI3K/Akt signaling pathway, alleviate neuronal damage, and improve cognitive impairment (Yi et al., 2019). LncRNA NON-HSAG045500 can regulate the expression of central neurotransmitter serotonin (5-HT) transporter, and it is a potential target for the treatment of major depressive disorder (Liu S. et al., 2018). Zhang et al. found that 68 lncRNAs were dysregulated in the postoperative cognitive dysfunction group compared to non-postoperative cognitive dysfunction group requiring hip or knee replacement surgery. Among them, the DE lncRNAs mainly participated in process of histone deacetylation (Zhang et al., 2018). In our study, we found that the expression of many lncRNAs was abnormal in patients of POD which results are consent with these published studies. However, we recognized different biological processes leading to the pathogenesis of POD. LncRNAs may regulate “glutamate and 5-HT”, “Syt7”, “TRP channel”, “IL-2 production”. If potential drugs regulating key lncRNAs are found, they may help reduce the occurrence of POD. Despite the promising findings, this study is just a system study for DE lncRNA and DE mRNA. Since the bioinformatics tools available to explore this hypothesis have limitations, this analysis need molecular biology experiments to verify. Finally, our study provides preliminary data on the mechanisms of lncRNAs in POD and future studies should include more molecular biology experiments to research the etiology of POD.

## Conclusion

In our study, the expression profile of lncRNAs and mRNAs in patients with POD was established by using microarray analysis and significantly DE lncRNAs and DE mRNAs were uncovered. We identified 14 significantly DE lncRNAs as candidate genes for further study. In this study, we found that lncRNAs play important regulatory roles in “glutamate and 5-HT”, “Syt7”, “TRP channel”, “IL-2 production” in POD. There was a regulatory relationship between lncRNA ENST00000530057 and Syt-7 mRNA. The mRNA level of PCLO was up-regulated in POD group. This study provides a new idea and direction for further study of the pathogenesis of POD and looking for potential therapeutic targets. It also provides greater data for other researchers.

## Data Availability Statement

The datasets presented in this study can be found in online repositories. The names of the repository/repositories and accession number(s) can be found below: https://www.ncbi.nlm.nih.gov/genbank/, GSE163943.

## Ethics Statement

The studies involving human participants were reviewed and approved by the Ethics Committee of Chinese PLA General Hospital (Beijing, China) (No. S2017-096-02). The patients/participants provided their written informed consent to participate in this study.

## Author Contributions

YS, JC, and WM conceived and planned the experiments. XW and AH performed the experiment and acquired the data. HL, JL, and YL analyzed and interpreted the data. YS drafted the manuscript. JC and WM revised the manuscript. All authors provided critical feedback and help in shaping the research, analysis, and manuscript. All authors have read and approved the final submitted manuscript.

## Conflict of Interest

The authors declare that the research was conducted in the absence of any commercial or financial relationships that could be construed as a potential conflict of interest.

## Footnotes

1 http://www.geneontology.org

2 https://www.kegg.jp/

3 https://string-db.org/

4 http://www.microrna.org/microrna/home.do/

5 http://www.targetscan.org/

## Supplementary Material

Supplementary Table 1 The primer sequences used for quantitative real-time PCR.

Supplementary Table 2 Detail information of Top 20 DE lncRNAs.

Supplementary Table 3 Detail information of Top 20 DE mRNAs.

Supplementary Table 4 Top30 GO terms of GO analysis in up-regulated DE mRNAs.

Supplementary Table 5 Top30 GO terms of GO analysis in down-regulated DE mRNAs.

Supplementary Table 6 Enriched pathways of KEGG analysis in up-regulated DE mRNAs.

Supplementary Table 7 Enriched pathways of KEGG analysis in down-regulated DE mRNAs.

## Abbreviations

LncRNAs: long non-coding RNAs
POD: postoperative delirium
DE: differentially expressed
RT-qPCR: quantitative reverse transcription PCR
GO: Gene Ontology
KEGG: Kyoto Encyclopedia of Genes and Genomes
PPI: Protein and protein interaction
CNC: coding and non-coding co-expression
ceRNA: competing endogenous RNA
CC: cellular component
MF: Molecular function
BP: Biological process
Syt: Synaptotagmin
CNS: Central nervous system
TRP: Transient receptor potential
IL: interleukin
PCLO: presynaptic cytomatrix protein
MCODE: Molecular Complex Detection
5-HT: 5-hydroxytryptamine.
