# Supplementary material for: Integrative Analysis of lncRNA and mRNA and Profiles in Postoperative Delirium Patients
Source: Front Aging Neurosci. 2021 May 19;13:665935. doi: 10.3389/fnagi.2021.665935 (PMC8171121; doi:10.3389/fnagi.2021.665935)
Supplement: Supplementary Table 1 — The primer sequences used for quantitative real-time PCR. [file Data_Sheet_1.docx]

Supplementary Material

**Table S1.** The primer sequences used for quantitative real-time PCR

| **Gene Name** | **Type** | **Sequence** | **Tm (℃)** | **Product Size (bp)** |
| --- | --- | --- | --- | --- |
| β-actin（H） | Internal control | F:5' GTGGCCGAGGACTTTGATTG3'  R:5’ CCTGTAACAACGCATCTCATATT3’ | 60 | 73 |
| NR_110435 | lncRNA | F:5' TCTCTCGAACTTCCTGTGTTAA 3’  R:5’ GCGTTGGGATTACCAGACA 3’ | 60 | 117 |
| T164670 | lncRNA | F:5' GGCTTCTGCCAAACACCAT 3’  R:5’ CCCTAGCAAGGAAACTTCGA 3’ | 60 | 199 |
| T270594 | lncRNA | F:5' AATAGTTCCCCAACAGTCAGC 3’  R:5’ ATCATCTCCTTGCACCATCA 3’ | 60 | 199 |
| ENST00000635048 | lncRNA | F:5' CCTCAACTTCCCAGGCTCAA 3’  R:5’ TGCTTTTTCCATTTTTTCCCAC 3’ | 60 | 216 |
| ENST00000452840 | lncRNA | F:5' GGGCATACATTACTTTCTTTCC 3’  R:5’ AAACAGGCAGTGGGGTAGAT 3’ | 60 | 101 |
| ENST00000426575 | lncRNA | F:5' GGACCCTGACATCATTTGC 3’  R:5’ TGGTGACAGCCCACTTTTAT 3’ | 60 | 128 |
| ENST00000513626 | lncRNA | F:5' CGCCTGTTTTTATGACTTGTTG 3’  R:5’ CCATTTCCAGTGAAAGCAGTTA 3’ | 60 | 267 |
| T206892 | lncRNA | F:5' TCATCCCTCAGTTGATCCTCA 3’  R:5’ TTTTGGTTCCCTGGTTTCTC 3’ | 60 | 201 |
| ENST00000562284 | lncRNA | F:5' TTCCAGGAGCTTGTGTCTGT 3’  R:5’ TTGTAAGGCATGTTGTCATCTT 3’ | 60 | 70 |
| ENCT00000461550 | lncRNA | F:5' TGTAGGAACACCCAAAAGTAAT 3’  R:5’ CTGGCTGTTTGTGTAGTATGAGT 3’ | 60 | 134 |
| T278393 | lncRNA | F:5' ATGGAACATCATCCTACACCG 3’  R:5’ GCTTTTTGAGTAGCCAGAGTTC 3’ | 60 | 64 |
| T379236 | lncRNA | F:5' CCAAGACATGCTGCAACCATT 3’  R:5’ TGCAGTCAGTACATCCCACA 3’ | 60 | 134 |
| ENST00000530057 | lncRNA | F:5' CCGAGTAGCTGGAATTACAGAC 3’  R:5’ GTGGCTTCTTCCTGAGCTTG 3’ | 60 | 148 |
| ENST00000609649 | lncRNA | F:5' CTTGTTCTAAACTGGGCTGGAG 3’  R:5’ CTGCTGCCCTGATGAAAAC 3’ | 60 | 85 |
| LINC01186 | lncRNA | F:5' CATGGCCTAGAGATGCAGAGG 3’  R:5’ GGGGCAGGTTCATGTCACAA 3’ | 60 | 230 |
| TCONS_00014317 | lncRNA | F:5' TCCTTTACAGGTGTGAGAGCCA 3’  R:5’ GAAACGGCGAGGCTTGGT 3’ | 60 | 157 |
| ENST00000637377 | lncRNA | F:5' CATAAGGTTCTGGTGGGGATT 3’  R:5’ GCAAAGTCTGTAAGGTGAGGGA 3’ | 60 | 157 |
| ENST00000421254 | lncRNA | F:5' GCATTGCCGACGAATCCTA 3’  R:5’ CAGGAGGTGAGCGAATGTCTA 3’ | 60 | 82 |
| ENST00000458677 | lncRNA | F:5' TTACAAAATGATACACAAAGACCT 3’  R:5’ CACAAAGCAACTAAAGAGGAGG 3’ | 60 | 75 |
| ENST00000448869 | lncRNA | F:5' CAAAGTCTCCCAGTCAATTCAG 3’  R:5’ TGGTCTAGGAATTTTGGAGAGG 3’ | 60 | 84 |
| DEFA4 | mRNA | F:5’ CCAGGCAAGAGGTGATGAGGCT3’  R:5’ ATGAGGCAGTTCCCAACACG3’ | 60 | 186 |
| PCLO | mRNA | F:5’ AAGCTGTGGATAAGGCGAAAT 3’  R:5’ CTGGGAACGGAACTGGATC3’ | 60 | 166 |
| NAMPT | mRNA | F:5’ TTCTGGAAACCCTCTTGACACT3’  R:5’ TGTTTCATGCCTTCTACAATC3’ | 60 | 171 |
| PON1 | mRNA | F:5’ TGTATTTGGGTTTAGCGTGGTC3’  R:5’ CATCGGGTGAAATGTTGATTCC3’ | 60 | 108 |
| NFRKB | mRNA | F:5’ GCCTGCCTACGTCACCATTCT3’  R:5’ GCTTTCGTCCAATGTCGTATT3’ | 60 | 218 |
| CAMTA2 | mRNA | F:5’ TTGGACTTAGAGCAGGAGGTTG3’  R:5’ AGGGTGGCGATAGGGCAAAT3’ | 60 | 265 |
| LTF | mRNA | F:5’ CGTAGGAGGAGTGTTCAGTG 3’  R:5’ AGGTCGCAGTTTGTAGGG 3’ | 60 | 225 |
| OR51E1 | mRNA | F:5’ AGTCTGCATAGGGCTTATAGCA3’  R:5’ CCCATGCCTAAGTCAGATTGC3’ | 60 | 254 |
| RGS11 | mRNA | F:5’ AAGTCCTCCGTCTGCCTTGA 3’  R:5’ TTGTCTGAGATCCAGGGATTGC3’ | 60 | 107 |
| LARP1B | mRNA | F:5’ AAGGCAGGAAAGCTCAAGACA3’  R:5’ CTTGGCTGAGGACGCTCTGA3’’ | 60 | 118 |
| LCE3E | mRNA | F:5’ CAGCCCCATCTGACGCATG3’  R:5’ GGGACATATGACATCCTGTACAT3’ | 60 | 135 |
| OR7A5 | mRNA | F:5’ TGTACCTGGTCACTGTGCTCG 3’  R:5’ TCAGCATTTTTGGAATGGTGGTG 3’ | 60 | 147 |
| BTN3A2 | mRNA | F:5’ TCCAAGATGGTGACTTCTATGA3’  R:5’ CCTCTCATGATCACAGATGC 3’ | 60 | 253 |
| USH1C | mRNA | F:5’ CCAGCCATTTGGGAGAAGATC 3’  R:5’ TTAGAGCAGAGGGCAGAGGAGA3’ | 60 | 163 |
| SCFD2 | mRNA | F:5’ TGGTTGCACCAGGCTGTCTT 3’  R:5’ ATGTCTCCGAACTTCCATCACT 3’ | 60 | 110 |
| KIAA1755 | mRNA | F:5’ GAGAAAGCCCACGCAGAATT3’  R:5’ CGCTGGGTAGAGGCAAAGG3’ | 60 | 170 |
| KNCN | mRNA | F:5’ TGCTCCCTGAACAAGCAAA3’  R:5’ AGGTGGCAAGACAGAGGTTGGT3’ | 60 | 174 |
| JMJD1C | mRNA | F:5’ GAGCCTCATCGGCCTCTTAA3’  R:5’ CAGACCGTAATGGTTCCATAAA 3’ | 60 | 118 |
| EIF5AL1 | mRNA | F:5’ GGTATGGAGGGTGTCTAGGTGT3’  R:5’ GTATTTTTCCTGCCACATGACTA 3’ | 60 | 170 |
| CIDEC | mRNA | F:5’ TGGCAGGGGATACAGTGTTCA3’  R:5’ TCTTCTTGGCAGGCTTATGGGAG3’ | 60 | 111 |

**Table S2.** Detail information of Top 20 DE lncRNAs

| Transcript ID | Gene ID | Gene Symbol | Fold Change | P-value | Regulation | Locus |
| --- | --- | --- | --- | --- | --- | --- |
| NR_110435 | ENSG00000224616 | RTCA-AS1 | 4.8529895 | 0.027822803 | up | chr1:100730298-100731730:- |
| NR_104061 | LINC00993 | LINC00993 | 4.3609112 | 0.011699659 | up | chr10:37598113-37635956:+ |
| T164670 | G038261 | G038261 | 4.2809819 | 0.003560136 | up | chr18:76423717-76430909:- |
| ENST00000440609 | ENSG00000225421 | AC019330.1 | 3.9649517 | 0.005796741 | up | chr2:199417919-199637080:- |
| compmerge.2410.pooled.chr16 | ENSG00000260528 | FAM157C | 3.6738362 | 0.042143783 | up | hg38:chr16:90106529-90120883+ |
| T270594 | G062727 | G062727 | 3.4657549 | 0.04932347 | up | chr4:122472825-122504338:+ |
| ENST00000635048 | ENSG00000226476 | LINC01748 | 3.4383103 | 0.043060482 | up | chr1:60985635-61061878:- |
| ENST00000425454 | ENSG00000230115 | TPRG1-AS2 | 3.373285 | 0.010535499 | up | chr3:188956475-188958383:- |
| ENST00000452840 | ENSG00000233723 | LINC01122 | 3.0347389 | 0.047140073 | up | chr2:58655473-59290901:+ |
| compmerge.1894.pooled.chr5 | ENSG00000249937 | LINC02223 | 2.9909222 | 0.008495424 | up | hg38:chr5:17807291-17929568+ |
| T278393 | G064585 | G064585 | 4.8204868 | 0.012768998 | down | chr5:5844821-5853715:+ |
| ENST00000619135 | ENSG00000234456 | MAGI2-AS3 | 3.7592294 | 0.017879653 | down | chr7:79083069-79088737:+ |
| T379236 | G089754 | G089754 | 3.6472239 | 0.02208173 | down | chrX:117820066-117821266:- |
| ENST00000530057 | ENSG00000149476 | TKFC | 3.3302004 | 0.028598773 | down | chr11:61100703-61105574:+ |
| ENST00000644419 | ENSG00000230426 | ERVMER61-1 | 3.1965926 | 0.003423872 | down | chr1:187276526-187456380:+ |
| ENST00000609649 | ENSG00000273416 | AL732292.2 | 3.1626365 | 0.013130565 | down | chr1:235267495-235267924:- |
| TCONS_00010009 | XLOC_004448 | XLOC_004448 | 3.0892571 | 0.003126583 | down | chr5:82683973-82694107:+ |
| ENST00000642737 | ENSG00000230426 | ERVMER61-1 | 3.0618049 | 0.002386561 | down | chr1:187197719-187299637:+ |
| ENST00000424415 | ENSG00000285756 | BX890604.2 | 3.0592125 | 0.010078475 | down | chrX:3809479-3820041:- |
| compmerge.2878.pooled.chrX | ENSG00000236751 | LINC01186 | 3.0388404 | 0.0195754 | down | hg38:chrX:46322668-46327652- |

**Table S3.** Detail information of Top 20 DE mRNAs

| Transcript_ID | Gene ID | Gene Symbol | Fold Change | *P*-value | Regulation | Locus |
| --- | --- | --- | --- | --- | --- | --- |
| ENST00000297435 | ENSG00000164821 | DEFA4 | 4.4543255 | 0.033014979 | up | chr8:6793344-6795860:- |
| ENST00000333891 | ENSG00000186472 | PCLO | 3.5910908 | 0.016164276 | up | chr7:82383329-82792246:- |
| ENST00000222553 | ENSG00000105835 | NAMPT | 3.5309288 | 0.034942355 | up | chr7:105888731-105925638:- |
| ENST00000222381 | ENSG00000005421 | PON1 | 3.2803645 | 0.045512793 | up | chr7:94926988-94954019:- |
| ENST00000524794 | ENSG00000170322 | NFRKB | 3.237338 | 0.01974862 | up | chr11:129734365-129762904:- |
| ENST00000414043 | ENSG00000108509 | CAMTA2 | 3.0311726 | 0.038395538 | up | chr17:4871292-4890407:- |
| ENST00000416284 | ENSG00000198673 | FAM19A2 | 3.0211613 | 0.013508811 | up | chr12:62102040-62586623:- |
| ENST00000231751 | ENSG00000012223 | LTF | 2.9236837 | 0.043291444 | up | chr3:46477136-46506653:- |
| ENST00000396952 | ENSG00000180785 | OR51E1 | 2.7808239 | 0.002802175 | up | chr11:4664650-4676718:+ |
| ENST00000377311 | ENSG00000181778 | TMEM252 | 2.653596 | 0.042239258 | up | chr9:71151496-71155783:- |
| MICT00000139456 | CATG00000032777 | CATG00000032777.1 | 4.1863136 | 0.012871024 | down | chr17:1651983-1657579:- |
| ENST00000594251 | ENSG00000268046 | AP001094.1 | 3.4973059 | 0.00712426 | down | chr18:8336437-8337038:- |
| ENST00000294600 | ENSG00000162592 | CCDC27 | 2.676682 | 0.003752539 | down | chr1:3668962-3688208:+ |
| ENST00000368789 | ENSG00000185966 | LCE3E | 2.6682917 | 0.014446495 | down | chr1:152538130-152539248:- |
| ENCT00000264922 | CATG00000054300 | CATG00000054300.1 | 2.5973632 | 0.020237371 | down | chr20:12596414-12598801:- |
| ENST00000322301 | ENSG00000188269 | OR7A5 | 2.5542469 | 0.030628182 | down | chr19:14935063-14946097:- |
| ENST00000358933 | ENSG00000038358 | EDC4 | 2.5284519 | 0.015037737 | down | chr16:67906926-67918406:+ |
| ENST00000527422 | ENSG00000186470 | BTN3A2 | 2.5255546 | 0.004577843 | down | chr6:26365459-26375965:+ |
| ENST00000447017 | ENSG00000163995 | ABLIM2 | 2.519193 | 0.033297723 | down | chr4:7967773-8160436:- |
| ENST00000005226 | ENSG00000006611 | USH1C | 2.5172434 | 0.030207902 | down | chr11:17515879-17565854:- |

**Table S4.** Top30 GO terms of GO analysis in up-regulated DE mRNAs

| Term | Ontology | Count | Pvalue | Enrichment.Score | GENES |
| --- | --- | --- | --- | --- | --- |
| myeloid leukocyte mediated immunity | Biological process | 21 | 5.47201E-06 | 5.26185337 | IL6//SPON2//FER//SERPINA3//CYBB//DEFA4//ALDH3B1//FCGR2A//CD93//COMMD3//LTF//MPO//DYNC1LI1//ATP6V1D//CALML5//CPPED1//RAB5B//MMP25//TRPM2//SIGLEC5//ADGRE5 |
| myeloid leukocyte activation | Biological process | 22 | 1.38833E-05 | 4.85750584 | JUN//TLR7//FER//CD93//SERPINA3//CYBB//DEFA4//ALDH3B1//FCGR2A//COMMD3//LTF//MPO//DYNC1LI1//ATP6V1D//CALML5//CPPED1//RAB5B//MMP25//TRPM2//SIGLEC5//ADGRE5//AGER |
| neutrophil mediated immunity | Biological process | 19 | 1.93922E-05 | 4.712372302 | SERPINA3//CYBB//DEFA4//ALDH3B1//FCGR2A//CD93//COMMD3//LTF//MPO//DYNC1LI1//ATP6V1D//CALML5//CPPED1//RAB5B//MMP25//TRPM2//SIGLEC5//ADGRE5//IL6 |
| neutrophil degranulation | Biological process | 18 | 4.39215E-05 | 4.357323084 | SERPINA3//CYBB//DEFA4//ALDH3B1//FCGR2A//CD93//COMMD3//LTF//MPO//DYNC1LI1//ATP6V1D//CALML5//CPPED1//RAB5B//MMP25//TRPM2//SIGLEC5//ADGRE5 |
| leukocyte degranulation | Biological process | 19 | 4.51439E-05 | 4.345400772 | FER//SERPINA3//CYBB//DEFA4//ALDH3B1//FCGR2A//CD93//COMMD3//LTF//MPO//DYNC1LI1//ATP6V1D//CALML5//CPPED1//RAB5B//MMP25//TRPM2//SIGLEC5//ADGRE5 |
| neutrophil activation involved in immune response | Biological process | 18 | 4.7567E-05 | 4.322694594 | SERPINA3//CYBB//DEFA4//ALDH3B1//FCGR2A//CD93//COMMD3//LTF//MPO//DYNC1LI1//ATP6V1D//CALML5//CPPED1//RAB5B//MMP25//TRPM2//SIGLEC5//ADGRE5 |
| myeloid cell activation involved in immune response | Biological process | 19 | 5.37793E-05 | 4.269384667 | FER//SERPINA3//CYBB//DEFA4//ALDH3B1//FCGR2A//CD93//COMMD3//LTF//MPO//DYNC1LI1//ATP6V1D//CALML5//CPPED1//RAB5B//MMP25//TRPM2//SIGLEC5//ADGRE5 |
| leukocyte mediated immunity | Biological process | 25 | 5.43434E-05 | 4.264853292 | AGER//IL6//SPON2//FZD5//CFI//FER//SERPINA3//CYBB//DEFA4//ALDH3B1//FCGR2A//CD93//COMMD3//LTF//MPO//DYNC1LI1//ATP6V1D//CALML5//CPPED1//RAB5B//MMP25//TRPM2//SIGLEC5//ADGRE5//NBN |
| neutrophil activation | Biological process | 18 | 6.17369E-05 | 4.209455193 | SERPINA3//CYBB//DEFA4//ALDH3B1//FCGR2A//CD93//COMMD3//LTF//MPO//DYNC1LI1//ATP6V1D//CALML5//CPPED1//RAB5B//MMP25//TRPM2//SIGLEC5//ADGRE5 |
| granulocyte activation | Biological process | 18 | 7.19306E-05 | 4.143086213 | SERPINA3//CYBB//DEFA4//ALDH3B1//FCGR2A//CD93//COMMD3//LTF//MPO//DYNC1LI1//ATP6V1D//CALML5//CPPED1//RAB5B//MMP25//TRPM2//SIGLEC5//ADGRE5 |
| secretory vesicle | Cellular component | 26 | 0.000264686 | 3.577269055 | LTF//MPO//KLK15//IGF1//SPINK14//SPAG9//DEFA4//PLA1A//ALDH3B1//FCGR2A//CD93//DYNC1LI1//RAB5B//SIGLEC5//ADGRE5//SERPINA3//SLC32A1//ADRA1D//SYT17//CYBB//ATP6V1D//MMP25//TRPM2//CPPED1//COMMD3//CALML5 |
| secretory granule | Cellular component | 23 | 0.000427249 | 3.369319453 | SPINK14//SPAG9//ALDH3B1//FCGR2A//CD93//DYNC1LI1//RAB5B//SIGLEC5//ADGRE5//SERPINA3//LTF//DEFA4//MPO//PLA1A//IGF1//CYBB//ATP6V1D//MMP25//TRPM2//CPPED1//COMMD3//CALML5//KLK15 |
| cytoplasmic vesicle part | Cellular component | 34 | 0.000440113 | 3.356435835 | TRPM2//LTF//COMMD3//CALML5//ALDH3B1//FCGR2A//CD93//DYNC1LI1//RAB5B//SIGLEC5//ADGRE5//ANTXR2//ACAP2//VPS41//TLR7//VPS37C//CD1A//RRAGD//SERPINA3//SLC32A1//ADRA1D//SYT17//PLA1A//CYBB//ATP6V1D//MMP25//FZD5//OSBPL11//CD300LG//RAB11FIP3//IGF1//MPO//CPPED1//DEFA4 |
| secretory granule membrane | Cellular component | 12 | 0.000492319 | 3.307753306 | PLA1A//CYBB//ALDH3B1//CD93//ATP6V1D//MMP25//TRPM2//SIGLEC5//DYNC1LI1//FCGR2A//RAB5B//ADGRE5 |
| bounding membrane of organelle | Cellular component | 43 | 0.000513957 | 3.289073372 | B3GALNT2//GCNT2//TLR7//ARFGAP1//CHST7//RAB2A//FZD5//COG8//B3GNT9//SLC33A1//SLC36A1//THBD//ALDH3B1//FCGR2A//CD93//DYNC1LI1//RAB5B//SIGLEC5//ADGRE5//ANTXR2//ACAP2//VPS41//VPS37C//CD1A//UMOD//PLA1A//CYBB//ATP6V1D//MMP25//TRPM2//OSBPL11//RAB11FIP3//PEX5L//MARC2//FLOT1//SLC32A1//ADRA1D//SYT17//CD300LG//RRAGD//LTF//SLC39A13//FAR2 |
| specific granule | Cellular component | 8 | 0.000985511 | 3.006338381 | CYBB//ALDH3B1//CD93//ATP6V1D//MMP25//TRPM2//DEFA4//LTF |
| specific granule membrane | Cellular component | 6 | 0.001034653 | 2.985205393 | CYBB//ALDH3B1//CD93//ATP6V1D//MMP25//TRPM2 |
| whole membrane | Cellular component | 35 | 0.001323496 | 2.878277249 | SLC36A1//THBD//ALDH3B1//FCGR2A//CD93//DYNC1LI1//RAB5B//SIGLEC5//ADGRE5//ANTXR2//ACAP2//VPS41//TLR7//VPS37C//CD1A//WIPI2//PLA1A//CYBB//ATP6V1D//MMP25//TRPM2//FZD5//OSBPL11//RAB11FIP3//PEX5L//MARC2//FLOT1//RAB2A//SLC32A1//ADRA1D//SYT17//CD300LG//RRAGD//RTN4RL1//FAR2 |
| anchored component of membrane | Cellular component | 7 | 0.004118748 | 2.385234786 | RTN4RL1//RAB5B//PRSS21//GML//SPRN//MMP25//UMOD |
| ficolin-1-rich granule membrane | Cellular component | 4 | 0.007358373 | 2.133218207 | CD93//DYNC1LI1//TRPM2//SIGLEC5 |
| solute:proton symporter activity | Molecular function | 3 | 0.003647142 | 2.438047301 | SLC36A1//SLC32A1//SLC33A1 |
| phosphatidylinositol-3,5-bisphosphate binding | Molecular function | 3 | 0.003647142 | 2.438047301 | ACAP2//WIPI2//PLEK2 |
| GDP binding | Molecular function | 4 | 0.006385378 | 2.194813401 | DYNC1LI1//RRAGD//RAB2A//RAB5B |
| IgG binding | Molecular function | 2 | 0.007987672 | 2.097579796 | FCGR2A//UMOD |
| S100 protein binding | Molecular function | 2 | 0.012893538 | 1.889627885 | AGER//FGF1 |
| vitamin D receptor binding | Molecular function | 2 | 0.014755429 | 1.831048168 | TAF11//THRAP3 |
| lipid binding | Molecular function | 16 | 0.016708026 | 1.777074847 | SPON2//LTF//UGT1A9//PON1//PCLO//SYT17//OSBPL2//CD1A//WIPI2//SNX31//PLEK2//UQCC3//ACAP2//OSBPL11//FER//FZD5 |
| insulin-like growth factor receptor binding | Molecular function | 2 | 0.018800795 | 1.725823785 | IGF1//INSL4 |
| amino acid:cation symporter activity | Molecular function | 2 | 0.028096417 | 1.551349053 | SLC36A1//SLC32A1 |
| scavenger receptor activity | Molecular function | 3 | 0.030195473 | 1.520058168 | AGER//CFI//HHIPL1 |

**Table S5**. Top30 GO terms of GO analysis in down-regulated DE mRNAs

| **Term** | **Ontology** | **Count** | **Pvalue** | **Enrichment.Score** | **GENES** |
| --- | --- | --- | --- | --- | --- |
| regulation of interleukin-2 production | Biological process | 7 | 6.49914E-05 | 4.187143956 | TBX21//PDE4B//PDE4D//RUNX1//CD3E//TRIM27//SPTBN1 |
| gamma-delta T cell activation | Biological process | 4 | 0.000105125 | 3.978295432 | NCKAP1L//SYK//NOD2//LILRB1 |
| interleukin-2 production | Biological process | 7 | 0.000152246 | 3.817453348 | TBX21//PDE4B//PDE4D//RUNX1//CD3E//TRIM27//SPTBN1 |
| positive regulation of interleukin-2 production | Biological process | 5 | 0.000272223 | 3.565075843 | CD3E//SPTBN1//PDE4B//PDE4D//RUNX1 |
| adenylate cyclase-inhibiting G-protein coupled receptor signaling pathway | Biological process | 8 | 0.000464582 | 3.332937732 | ADCY5//GRM2//CHRM2//HTR1B//ADCY6//ADORA1//LPAR1//GNAZ |
| serotonin secretion | Biological process | 3 | 0.000907292 | 3.042252763 | SYK//LILRB1//HTR1B |
| cellular component assembly | Biological process | 81 | 0.002153409 | 2.666873566 | MAP1LC3B//KAT6B//USH1C//FSCN2//ERCC2//UBN1//CELF4//NCK1//TUBGCP2//P3H4//ADRB2//CD3E//FKBP1A//ZFYVE9//NEURL1//SPTBN2//COBLL1//FOPNL//WDR60//CAND2//HSCB//GJB1//NCKAP1L//PDGFA//ABLIM2//PLD6//HIP1R//CDC42EP1//TNRC18//KRT5//KRT14//LPAR1//TRABD2A//COX14//CIRBP//TRIM27//GMFG//SOAT2//APOA5//SDHAF4//SDHAF1//RSPH9//JHY//IFT27//KCNG3//TOR2A//KCNA10//SHKBP1//PSMG2//E2F4//KRT19//TBC1D30//PIP5K1A//SEC23A//MCFD2//AP2S1//SHMT2//APP//RCC1//NOD2//FHOD1//SPTBN1//LRRC24//HNF1B//VAMP2//TSPAN4//SKAP2//ASIC1//CRK//SH3PXD2A//CDC20//SYT7//SMARCAL1//ODF2//HYAL1//RPL13A//CEP295//PHLDB1//RTEL1//POC1A//RUNX1 |
| adenylate cyclase-modulating G-protein coupled receptor signaling pathway | Biological process | 11 | 0.002647646 | 2.577140105 | ADCY5//ADCY6//ADM2//ADORA1//LPAR1//GNAZ//HTR1B//GRM2//CHRM2//ADRB2//PDE4D |
| epidermal cell differentiation | Biological process | 16 | 0.002681681 | 2.571592908 | DNASE1L2//LCE1A//LCE1E//LCE3E//PIP5K1A//KRT5//KRT14//KRT19//KRTAP19-8//KRTAP17-1//SPINK5//ERCC2//PITX2//USH1C//RUNX1//KLK14 |
| serotonin transport | Biological process | 3 | 0.003189216 | 2.49631606 | SYK//LILRB1//HTR1B |
| coated vesicle | Cellular component | 15 | 0.000484866 | 3.314377834 | APP//LMAN1L//RGS19//PHETA1//VAMP2//HIP1R//RAB8B//AP2S1//CTLA4//SEC23A//MCFD2//CHRM2//ADRB2//COPZ2//EPN2 |
| actin-based cell projection | Cellular component | 11 | 0.002160051 | 2.665535898 | USH1C//PDGFA//WWOX//PLEKHG6//GRXCR2//CD302//KITLG//ENAH//FSCN2//TSPEAR//APP |
| clathrin coat of endocytic vesicle | Cellular component | 3 | 0.003043481 | 2.516629378 | AP2S1//HIP1R//EPN2 |
| cytoplasm | Cellular component | 257 | 0.003359297 | 2.473751658 | SPINK5//MYH10//HIP1R//CYTIP//CD302//PHLDB1//KIF1B//UCHL1//FKBP1A//P3H2//MOV10L1//RSPH9//MAP1LC3B//GOLT1A//SEC23A//DHCR24//FNDC3A//GALNT9//COPZ2//SCARA3//PDGFA//XYLT1//GALNT14//LMAN1L//HS6ST2//MCFD2//NCK1//RPL13A//WDR60//ZFYVE1//C6ORF106//TM9SF1//PRKD1//SYVN1//TUBGCP2//PDE4B//SAMD4A//EDC4//DNAJC11//AKAP3//SPINK13//ADRB2//HYAL1//LAMP2//SYT7//SHKBP1//C19ORF70//NADK2//SDHAF4//AIFM3//HSCB//CYP11A1//E2F1//ABCD1//VWA8//GLRX//KRT5//CHCHD10//LAP3//YARS2//WWOX//RAB8B//PHYH//TOMM7//ENOSF1//NOD2//SDHAF1//SHMT2//TNRC18//COX14//KIAA0391//MAN2C1//USH1C//SRRM1//MYL9//ZNF268//AP2S1//TBC1D16//CRK//ASB5//PHETA1//DAO//GIMAP7//ERCC2//EPN2//ARHGAP26//TBC1D30//GCA//RCHY1//GNAZ//GPI//N6AMT1//FHOD1//NCKAP1L//APP//LCE1A//INPP4A//IREB2//KRT14//KRT19//FEZF1//MAGEA10//NFX1//NPAS2//ODF2//ANO7//PDE4D//PLCD1//PLCG1//RIN2//RABL6//ENAH//PSMG2//TRIM27//AGBL5//SBF1//CIDEC//NXN//SGTA//COPS7B//SPTBN1//SPTBN2//VAMP2//SYK//TNNI2//KRTAP19-8//UCK2//PDZD3//BORA//AAAS//KDM5C//KRTAP17-1//PIP5K1A//POLR3GL//SPSB2//AGBL4//PPFIBP1//CEP295//KHSRP//RUNX1//SPHK1//SKAP2//RNF8//ATP23//DEPDC7//UBE2L6//ZFYVE9//AKAP7//SH3PXD2A//SERTAD2//CDC20//TMEM173//RPS6KL1//PLD6//TSPO2//ARMCX2//SLC25A35//SLC25A51//ACSM4//BORCS8//CD63//IFT27//LPAR1//ATP9B//RAB25//P3H4//ADORA1//KCNG3//FAIM2//MGST3//CBLN3//ULBP2//MPIG6B//SOAT2//DERL3//APOA5//TOR2A//EOGT//APLP2//LTBP1//COL18A1//EIF5AL1//ANO5//DDN//GJB1//ATP13A1//PCYT2//RGS19//CTLA4//ASIC1//CPZ//ACAN//AGRP//RBFOX1//KLK14//SCG5//FOPNL//POC1A//ADCY5//ADCY6//CIRBP//AP4S1//CHRM2//STAB1//APOH//PDLIM4//KNCN//GMFG//NFAM1//SERPINF1//TLK2//MAP3K4//TMEM134//NEURL1//CASP5//MTRNR2L1//MTRNR2L10//MTRNR2L11//LRRC3C//MYBBP1A//LILRB1//RCC1//PTPN21//CDC42EP1//SGCZ//DQX1//DNASE1L2//DUSP5//ELF5//NRK//MAPK15//GABRB1//FSCN2//SENP3//SENP1//HTR1B//NLRP10//RBPMS2//LCE1E//LCE3E//ISG20//KITLG//PITX2//YTHDF1//UBR7//PLEKHG6//CELF4//BEGAIN//PVALB//ZIC2//ZIC3//SVEP1//SH3BP5L//ABLIM2//BEX2//GAS7//UNK |
| clathrin-coated vesicle | Cellular component | 10 | 0.003371452 | 2.472182966 | RAB8B//CHRM2//ADRB2//VAMP2//HIP1R//AP2S1//CTLA4//EPN2//RGS19//PHETA1 |
| autophagosome membrane | Cellular component | 4 | 0.00367315 | 2.434961368 | LAMP2//TM9SF1//PRKD1//MAP1LC3B |
| dendritic spine | Cellular component | 9 | 0.003785068 | 2.421926263 | DDN//ADORA1//LPAR1//APP//PDE4B//PDLIM4//HIP1R//NEURL1//CD3E |
| intracellular part | Cellular component | 310 | 0.004058577 | 2.391626209 | RCHY1//RNF8//KHSRP//RGS19//GNAZ//GNG8//PSMG2//MTRNR2L1//MTRNR2L10//MTRNR2L11//LRRC3C//USH1C//MYBBP1A//AKAP3//ZNF268//LILRB1//SPINK5//RCC1//PTPN21//CDC42EP1//CIRBP//SGCZ//CRK//KNCN//HSCB//DQX1//DHCR24//DNASE1L2//DUSP5//ELF5//NRK//ERCC2//ABCD1//MAPK15//FKBP1A//DDN//RPL13A//EDC4//GABRB1//FSCN2//GCA//SENP3//N6AMT1//FHOD1//SENP1//HTR1B//HYAL1//NLRP10//RBPMS2//APP//LCE1A//LCE1E//LCE3E//INPP4A//IREB2//ISG20//KRT5//KRT14//MAP3K4//KITLG//MYH10//NCK1//WWOX//PITX2//PLCD1//PLCG1//RBFOX1//YTHDF1//UBR7//PLEKHG6//RABL6//CELF4//BEGAIN//PVALB//TRIM27//AGBL5//SBF1//NOD2//NXN//SGTA//SHMT2//SPTBN1//SYK//UCHL1//ZIC2//ZIC3//SVEP1//SH3BP5L//RPS6KL1//CASP5//SYVN1//ABLIM2//BEX2//GAS7//UNK//CEP295//PDLIM4//SPHK1//SKAP2//HIP1R//CYTIP//SERTAD2//E2F4//NHLH2//NPAS2//TBX2//HNF1B//TSPO2//EME1//C19ORF70//AIFM3//CYP11A1//SLC25A35//SLC25A51//DAO//PLD6//TMEM173//TOMM7//ARMCX2//AP4S1//GIMAP7//EPN2//MGST3//RAB8B//ATP13A1//ADAMTSL3//SMARCD1//VAMP2//RUNX1//ATP23//ZFYVE9//ZFYVE1//C6ORF106//RSPH9//MAP1LC3B//NADK2//SDHAF4//E2F1//VWA8//KIF1B//GLRX//CHCHD10//LAP3//YARS2//PHYH//ENOSF1//DNAJC11//SDHAF1//TNRC18//COX14//KIAA0391//MAN2C1//SRRM1//MYL9//SEC23A//TUBGCP2//AP2S1//TBC1D16//ASB5//PHETA1//FNDC3A//SAMD4A//ARHGAP26//TBC1D30//GPI//NCKAP1L//KRT19//FEZF1//MAGEA10//NFX1//ODF2//ANO7//COPZ2//PDE4B//PDE4D//RIN2//ENAH//PRKD1//CIDEC//COPS7B//SPTBN2//TNNI2//KRTAP19-8//UCK2//PDZD3//BORA//AAAS//KDM5C//KRTAP17-1//PIP5K1A//POLR3GL//SPSB2//AGBL4//PPFIBP1//SYT7//DEPDC7//UBE2L6//AKAP7//SH3PXD2A//CDC20//P3H4//GOLT1A//ADORA1//KCNG3//FAIM2//SCARA3//P3H2//CBLN3//ULBP2//MPIG6B//SOAT2//LMAN1L//IFT27//CTLA4//ASIC1//GALNT9//GALNT14//CPZ//TM9SF1//RAB25//MCFD2//TLK2//ATP9B//SERPINF1//TMEM134//NEURL1//DERL3//ACSM4//APOA5//TOR2A//EOGT//APLP2//LTBP1//PDGFA//COL18A1//ACAN//AGRP//CD63//NARF//GMFG//KAT6B//CBX8//ADRB2//LAMP2//SHKBP1//CD302//EGLN2//FAM71E1//FOPNL//CSRP2//ZNF782//TMEM92//ARX//JMJD1C//FERD3L//RALY//CAND2//TIPARP//GBX1//ZNF575//UHRF1//UBN1//TBX21//FAM170A//MGMT//ATF3//ODF1//ORC2//SMARCAL1//ZNF771//RTEL1//NSD3//TCEAL7//REXO4//ZNF16//PBX4//L3MBTL2//C2ORF16//EIF1AD//FAM220A//KLF7//MDC1//LPAR1//KLK14//SCG5//MOV10L1//BCAS2//DEDD2//POC1A//HOXC8//ADCY5//FAM9B//EIF5AL1//SCAF8//HOXD1//PRDM14//HS6ST2//WDR60//XYLT1//ANO5//GJB1//PCYT2//STAB1//ADCY6//BORCS8//PHLDB1//APOH//NFAM1//SPINK13//CHRM2 |
| stereocilium bundle | Cellular component | 5 | 0.00411801 | 2.385312652 | USH1C//FSCN2//TSPEAR//GRXCR2//KNCN |
| neuron spine | Cellular component | 9 | 0.004122473 | 2.384842189 | ADORA1//LPAR1//APP//PDE4B//PDLIM4//HIP1R//NEURL1//CD3E//DDN |
| translation repressor activity | Molecular function | 4 | 0.001457673 | 2.83633997 | CELF4//CIRBP//SAMD4A//IREB2 |
| translation regulator activity | Molecular function | 5 | 0.004166356 | 2.380243605 | CELF4//CIRBP//SAMD4A//IREB2//NEURL1 |
| scaffold protein binding | Molecular function | 5 | 0.006592719 | 2.180935414 | ADCY5//CRK//KIF1B//KRT5//PDE4D |
| L-ascorbic acid binding | Molecular function | 3 | 0.007136888 | 2.146491139 | EGLN2//PHYH//P3H2 |
| iron-sulfur cluster binding | Molecular function | 5 | 0.009258493 | 2.033459716 | AIFM3//ERCC2//IREB2//RTEL1//NARF |
| metal cluster binding | Molecular function | 5 | 0.009258493 | 2.033459716 | NARF//AIFM3//ERCC2//IREB2//RTEL1 |
| phosphoric diester hydrolase activity | Molecular function | 6 | 0.010114136 | 1.995071229 | PDE4D//PDE4B//PLCD1//PLCG1//ADORA1//PLD6 |
| anion binding | Molecular function | 72 | 0.010259128 | 1.988889536 | SOAT2//PLCD1//SYT7//TLK2//ADCY5//ADCY6//NADK2//DQX1//NRK//ERCC2//ABCD1//MAPK15//VWA8//KIF1B//TOR2A//NLRP10//ACSM4//ATP9B//IQCA1L//MAP3K4//MYH10//SMARCAL1//YARS2//RTEL1//MOV10L1//DALRD3//ABCF3//PRKD1//ATP13A1//NOD2//SYK//UCK2//RPS6KL1//PIP5K1A//RUNX1//SPHK1//IFT27//GIMAP7//GNAZ//RAB8B//RABL6//RAB25//SCG5//ACAN//STAB1//APOA5//ARHGAP26//APOH//SPTBN1//SPTBN2//VAMP2//ZFYVE1//ZFYVE9//HIP1R//COMP//APLP2//APP//MPIG6B//WISP2//SHMT2//PDE4B//PDE4D//PHYH//EGLN2//P3H2//SH3PXD2A//TMEM173//LPAR1//RCC1//AIFM3//DHCR24//DAO |
| receptor signaling complex scaffold activity | Molecular function | 3 | 0.01194304 | 1.922885111 | DEDD2//NCK1//CD3E |
| glycosaminoglycan binding | Molecular function | 10 | 0.011956598 | 1.922392355 | ACAN//STAB1//APOA5//COMP//APLP2//APOH//APP//MPIG6B//WISP2//NOD2 |
|  |  |  |  |  |  |
|  |  |  |  |  |  |

**Table S6.** Enriched pathways of KEGG analysis in up-regulated DE mRNAs

| Definition | Fisher-Pvalue | Selection Counts | Enrichment_Score | Genes |
| --- | --- | --- | --- | --- |
| AGE-RAGE signaling pathway in diabetic complications - Homo sapiens (human) | 0.000460561 | 7 | 3.336713 | AGER//COL4A2//COL4A5//CYBB//IL6//JUN//THBD |
| NOD-like receptor signaling pathway - Homo sapiens (human) | 0.003631731 | 8 | 2.439886 | ANTXR2//CYBB//DEFA4//IL6//JUN//NAMPT//NLRP7//TRPM2 |
| Amoebiasis - Homo sapiens (human) | 0.01363392 | 5 | 1.865379 | CD1A//COL4A2//COL4A5//IL6//RAB5B |
| Phagosome - Homo sapiens (human) | 0.0190715 | 6 | 1.719615 | ATP6V1D//CYBB//DYNC1LI1//FCGR2A//MPO//RAB5B |
| Hypertrophic cardiomyopathy (HCM) - Homo sapiens (human) | 0.03640668 | 4 | 1.438819 | CACNG5//IGF1//IL6//ITGA9 |
| Histidine metabolism - Homo sapiens (human) | 0.04013465 | 2 | 1.39648 | ALDH3B1//HAL |

**Table S7.** Enriched pathways of KEGG analysis in down-regulated DE mRNAs

| **Definition** | **Fisher-Pvalue** | **Selection Counts** | **Enrichment_Score** | **Genes** |
| --- | --- | --- | --- | --- |
| Phospholipase D signaling pathway - Homo sapiens (human) | 0.000195788 | 10 | 3.708213 | ADCY5//ADCY6//GRM2//KITLG//LPAR1//PDGFA//PIP5K1A//PLCG1//SPHK1//SYK |
| Morphine addiction - Homo sapiens (human) | 0.000851502 | 7 | 3.069815 | ADCY5//ADCY6//ADORA1//GABRB1//GNG8//PDE4B//PDE4D |
| Regulation of actin cytoskeleton - Homo sapiens (human) | 0.003245588 | 10 | 2.488707 | BUB1B-PAK6//CHRM2//CRK//ENAH//LPAR1//MYH10//MYL9//NCKAP1L//PDGFA//PIP5K1A |
| Rap1 signaling pathway - Homo sapiens (human) | 0.009224452 | 9 | 2.035059 | ADCY5//ADCY6//CRK//ENAH//KITLG//LPAR1//PDGFA//PLCG1//PRKD1 |
| cAMP signaling pathway - Homo sapiens (human) | 0.01097463 | 9 | 1.95961 | ADCY5//ADCY6//ADORA1//ADRB2//CHRM2//HTR1B//MYL9//PDE4B//PDE4D |
| Regulation of lipolysis in adipocytes - Homo sapiens (human) | 0.01381155 | 4 | 1.859757 | ADCY5//ADCY6//ADORA1//ADRB2 |
| Fc gamma R-mediated phagocytosis - Homo sapiens (human) | 0.02056683 | 5 | 1.686833 | CRK//PIP5K1A//PLCG1//SPHK1//SYK |
| Renin secretion - Homo sapiens (human) | 0.02924067 | 4 | 1.534013 | ADCY5//ADCY6//ADORA1//ADRB2 |
| T cell receptor signaling pathway - Homo sapiens (human) | 0.03034306 | 5 | 1.517941 | BUB1B-PAK6//CD3E//CTLA4//NCK1//PLCG1 |
| Inositol phosphate metabolism - Homo sapiens (human) | 0.03650781 | 4 | 1.437614 | INPP4A//PIP5K1A//PLCD1//PLCG1 |
| Steroid biosynthesis - Homo sapiens (human) | 0.04039418 | 2 | 1.393681 | DHCR24//SOAT2 |
| Serotonergic synapse - Homo sapiens (human) | 0.04541064 | 5 | 1.342842 | ADCY5//APP//GABRB1//GNG8//HTR1B |
| Ovarian steroidogenesis - Homo sapiens (human) | 0.04985375 | 3 | 1.302302 | ADCY5//ADCY6//CYP11A1 |
